# Potential Corneal Donation in Patients with Out-of-hospital Cardiac Arrest: A Case-control Study

**DOI:** 10.31662/jmaj.2024-0380

**Published:** 2025-06-20

**Authors:** Yuji Okazaki, Noritomo Fujisaki, Hideto Fukui, Kyungko Huh, Takayuki Otani, Toshihisa Ichiba, Hiroshi Naito, Yuki Kataoka

**Affiliations:** 1 Department of Emergency Medicine, Hiroshima City Hiroshima Citizens Hospital, Hiroshima, Japan; 2 Scientific Research WorkS Peer Support Group (SRWS-PSG), Osaka, Japan; 3 Fukui Medical Clinic, Hiroshima, Japan; 4 Department of Internal Medicine, Kyoto Min-iren Asukai Hospital, Kyoto, Japan; 5 Section of Clinical Epidemiology, Department of Community Medicine, Kyoto University Graduate School of Medicine, Kyoto, Japan; 6 Department of Healthcare Epidemiology, Kyoto University Graduate School of Medicine / Public Health, Kyoto, Japan

**Keywords:** case-control study, corneal donation, emergency department, out-of-hospital cardiac arrest, potential donor

## Abstract

**Introduction:**

The shortage of corneal donations has been a longstanding problem in Japan. However, there are limited data on the settings in which corneal donations occur. The aim of this study was to determine the association between out-of-hospital cardiac arrest (OHCA) and corneal donation. We also investigated potential corneal donors across various death settings (OHCA, in-hospital cardiac arrest (IHCA), and home death).

**Methods:**

This was a case-control study in Hiroshima Prefecture, Japan, from 2015 to 2023. Cases included all corneal donors based on data provided by the Hiroshima Eye Bank, while controls consisted of potential corneal donors aged ≥10 years at Hiroshima City Hiroshima Citizens Hospital. After matching cases with controls at a 1:5 ratio using age group, sex, and date of death, we employed unconditional logistic regression to analyze the association between OHCA and corneal donation.

**Results:**

From the combined data of the Hiroshima Eye Bank (n = 190), the hospital cohort (n = 4492), and the home cohort (n = 195), 190 were corneal donors and 3,394 were potential corneal donors. Among corneal donors, 5.3% (10/190) had OHCA, 26% (49/190) had IHCA, and 29% (55/190) died at home or in nursing homes. Among the various death settings, 72% (2,768/3,835) in the hospital cohort and 69% (134/195) in the home cohort were potential corneal donors, with OHCA patients in the hospital cohort showing the highest proportion (75%, 492/657). After adjusting for matching factors, OHCA was positively associated with corneal donation (13% [26/190] vs 15% [143/950]; odds ratio: 2.2, 95% confidence interval: 1.3-3.5, p = 0.0019).

**Conclusions:**

Individuals who experienced OHCA may be more likely than individuals in other settings of death to become corneal donors. Further research is needed to confirm this study’s findings and to explore strategies to address the issue of Japan’s corneal donation shortage.

## Introduction

Corneal transplantation in Japan officially began in 1958 with government approval, and eye banks have been established in each prefecture of Japan ^(1)^. The number of domestic corneal donations in Japan has not increased over the past decade, and the number of patients waiting for corneal transplants has exceeded the number of available donor corneas within Japan during this period ^(2), (3)^. To address this issue, Japan has relied on imported corneas from overseas to perform corneal transplants ^(3)^. The Istanbul Declaration states that countries should strive to be self-sufficient in meeting their own needs, and measures should be taken to increase domestic corneal donations in Japan ^(4)^. For many years, eye banks and awareness-raising organizations have been making various efforts to solve the problem of a shortage of corneal donations, but there has still not been a sufficient increase in corneal donations.

Potential corneal donors include patients who experience out-of-hospital cardiac arrest (OHCA). In Japan, approximately 120,000 people are transported to hospitals annually by ambulance due to OHCA, and most of them are taken to tertiary hospitals ^(5)^. However, it is often difficult to achieve return of spontaneous circulation (ROSC), resulting in the inability to save the lives of these patients ^(5)^. Criteria for corneal donation include a time limit of 6 hours from cardiac arrest to corneal donation, absence of sepsis, and no history of certain viral infections. The majority of patients transported to hospitals due to OHCA may meet these criteria. In other words, patients transported to hospitals due to OHCA have the potential to be corneal donors. However, no study has been conducted in Japan to clarify the patient groups and settings in which corneal donations are performed. Little is known about the association between corneal donations and settings of death (i.e., OHCA, in-hospital cardiac arrest (IHCA), and home death).

The purpose of this study was to examine the association between patients who experienced OHCA and corneal donation. We also examined the extent to which potential donors exist among different settings of death.

## Materials and Methods

### Study design and settings

We conducted a case-control study from January 1, 2015, to December 31, 2023, in Hiroshima Prefecture, Japan. We used data from Hiroshima Eye Bank in Hiroshima Prefecture, and from the electronic charts of Hiroshima City Hiroshima Citizens Hospital and Fukui Internal Medicine Clinic. In 2023, Hiroshima Prefecture had a population of 2.7 million, the 11th largest in Japan, and an aging rate of 29.7%. Hiroshima City, where Hiroshima City Hiroshima Citizens Hospital and Fukui Internal Medicine Clinic are located, had a population of 1.18 million and an aging rate of 26% ^(6)^. Hiroshima City Hiroshima Citizens Hospital, located in the center of Hiroshima City, is a tertiary care institution that receives approximately 200 cases of OHCA per year. Fukui Internal Medicine Clinic is a home care support clinic that provides palliative home care, with approximately 50 home deaths per year. Data from Fukui Internal Medicine Clinic were available from January 1, 2020, to December 31, 2023. The protocol of this study was approved by the Institutional Review Board of Hiroshima City Hiroshima Citizens Hospital (approval number: 2024-35). Due to the use of anonymized data and the adoption of an opt-out approach for participant enrollment, the requirement for informed consent was waived. To report this study, we followed the Strengthening the Reporting of Observational Studies in Epidemiology statement (Supplementary Table S1) ^(7)^.

### Selection and matching of cases and controls

From the dataset of Hiroshima Eye Bank, we included all individuals who donated their corneas during the study period in Hiroshima Prefecture as cases. We did not exclude individuals who donated their corneas under brain death or cardiac arrest. As controls, we selected potential corneal donors aged ≥10 years among all individuals who died in Hiroshima City, Hiroshima Citizens Hospital (Hospital cohort). We defined potential corneal donors as individuals who met the criteria of corneal donation in Japan but did not donate corneas. The criteria are shown in Supplementary Table S2. We referred to the case group as the corneal donation group and the control group as the potential donation group. We matched the case group to the control group at a target ratio of 1:5 ^(8), (9)^. We included age group, sex, and date of death as matching factors. Age groups were categorized as 10-19, 20-29, 30-39, 40-49, 50-59, 60-69, 70-79, 80-89, 90-99, and 100 years of age or older. Dates of death were divided into two groups: pre-coronavirus disease 2019 (COVID-19) period (from January 2015 to December 2019) and post-COVID-19 period (from January 2020 to December 2023). We used these two groups as a matching factor.

### Definition of exposure

We defined OHCA as Emergency Medical Service (EMS)-transported out-of-hospital cardiac arrest with the declaration of death at an emergency department (ED). We defined in-hospital cardiac arrest (IHCA) as cardiac arrest in individuals who occupied an inpatient hospital bed in our hospital ^(10)^. Individuals who had ROSC after EMS-transported OHCA but later died after admission were included as individuals with IHCA. For individuals who donated their corneas, cases in which we could not determine whether OHCA had occurred because private information, such as detailed reasons for death and the names of the place where corneal donation was conducted, was not available, were treated as cases with missing data. Based on available data from the Hiroshima Eye Bank, we categorized the subjects who donated their corneas into subjects with OHCA, those with IHCA, and those who died at home or in a nursing home. In the hospital cohort, we categorized subjects who died in our hospital into subjects with OHCA and subjects with IHCA. There were no subjects who experienced OHCA in the home cohort.

### Outcomes

To clarify the association between OHCA and corneal donation in Hiroshima Prefecture, we compared the proportion of subjects with OHCA among corneal donors and the proportion of subjects with OHCA among potential corneal donors. In addition, we examined potential corneal donors among all subjects across different settings of death.

### Statistical analysis

Dichotomous variables and categorical variables were summarized using percentages. We calculated the proportion of potential corneal donors among three groups: 1) subjects who experienced OHCA in our hospital, 2) subjects who experienced IHCA in our hospital, and 3) subjects who died at home under the care of Fukui Internal Medicine Clinic. Differences in proportions among these groups were compared using the χ^2^-test. We could not calculate the sample size because there were no previous studies that investigated the relationship between corneal donation and the circumstances of death. All available data were therefore used for the analysis. We handled missing data under the missing-at-random assumption using multiple imputation. We incorporated all covariates (i.e., age group, sex, date of death, settings of death, and corneal donation) and exposures into the imputation model. We generated 100 imputed datasets and analyzed each dataset separately using the analysis model ^(11)^. The results from the 100 datasets were then combined using Rubin’s rules to obtain the final estimates and their standard errors ^(12)^. To compare the proportions of subjects who experienced OHCA in the corneal donation and potential donation groups, we employed an unconditional logistic regression model ^(9)^. Cases and controls were individually matched on age group, sex, and date of death. We included these variables as covariates in the regression model because matching in case-control studies does not itself eliminate confounding by the matching factors and can potentially introduce confounding even when it did not exist in the source population ^(13)^. To confirm the robustness of the main analysis, we conducted a sensitivity analysis using a conditional logistic regression model that adjusted for the matching factors (i.e., age group, sex, and date of death). This analysis was performed to test the assumption that adjustment for matching factors in an unconditional logistic regression model adequately controls for potential confounding introduced by the matching process. We reported effect sizes as odds ratios (ORs) with 95% confidence intervals (CIs) and two-sided p-values. We performed statistical analyses using R software (version 4.3.2; R Foundation for Statistical Computing, Vienna, Austria).

## Results

### Characteristics of corneal donation and potential donation groups

**Figure 1.**
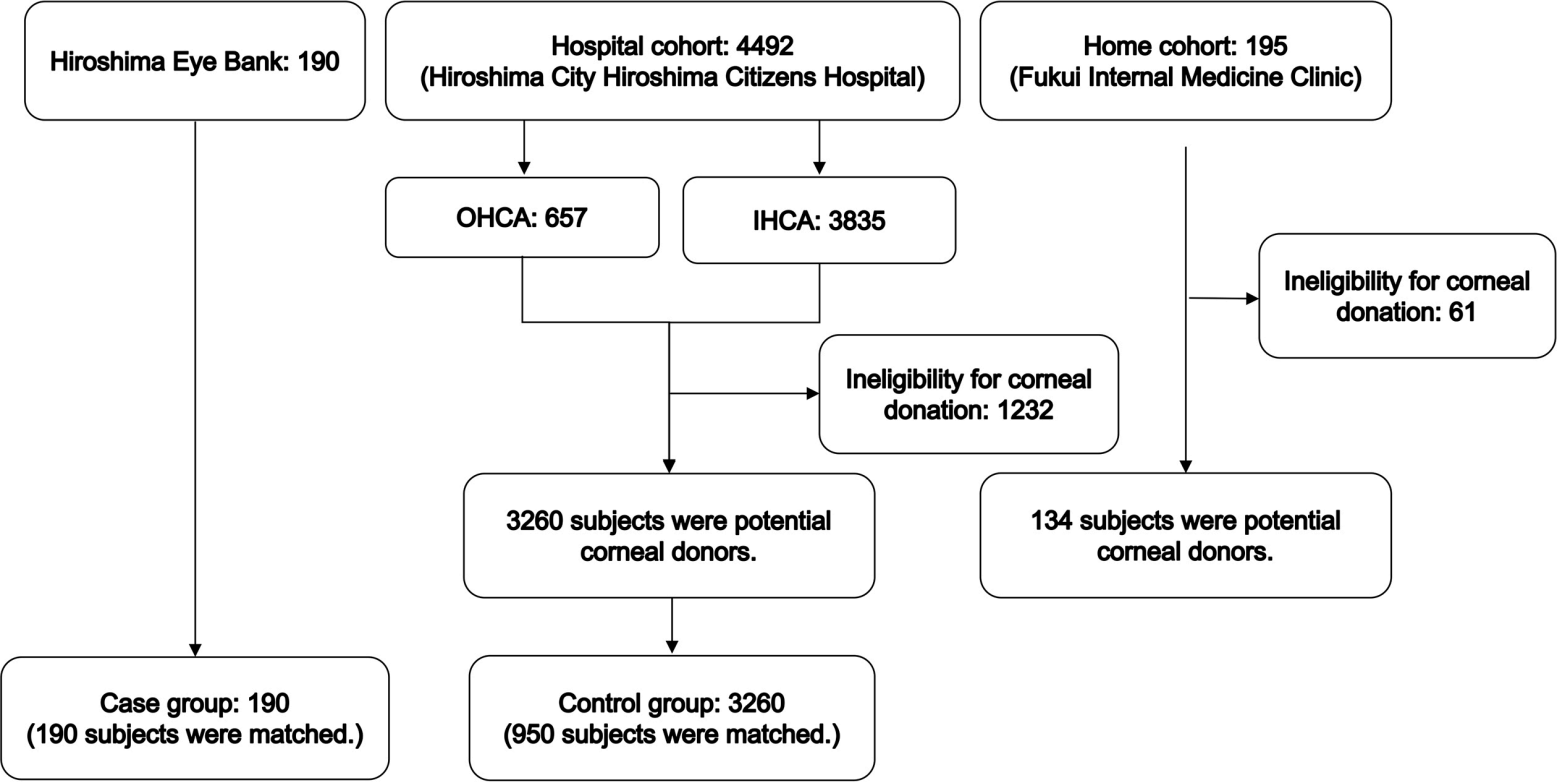
Subject flow chart in the case and control groups. IHCA: in-hospital cardiac arrest; OHCA: out-of-hospital cardiac arrest.

We identified 190 subjects from data in the Hiroshima Eye Bank, 4,492 subjects in the hospital cohort, and 195 subjects in the home cohort (Figure 1). Table 1 shows the characteristics of subjects in the corneal donation and potential donation groups. We included 190 subjects with corneal donation as the corneal donation group and 3,394 subjects with potential corneal donation as the potential donation group: 3,260 subjects in the hospital cohort and 134 subjects in the home cohort. During the observation period, approximately 20 subjects donated their corneas annually. In the corneal donation group, 10 subjects (5.3%) with OHCA donated their corneas, 49 subjects (26%) with IHCA donated their corneas, and 55 subjects (29%) who died at home or in a nursing home donated their corneas. In the hospital cohort of the potential donation group, 492 subjects (15%) had OHCA and 2,768 subjects (85%) had IHCA. In the home cohort, 134 subjects (100%) died at home.

**Table 1.** Characteristics of Subjects with Corneal Donations and Potential Corneal Donors.

	Case: corneal donation (n = 190)	Control: Potential corneal donor*	
		Hospital cohort (n = 3,260)	Home cohort (n = 134)
Date (year), n (%)			
2015	23 (12)	396 (12)	-
2016	29 (15)	426 (13)	-
2017	16 (8.4)	421 (13)	-
2018	21 (11)	389 (12)	-
2019	19 (10)	331 (10)	-
2020	21 (11)	333 (10)	15 (11)
2021	18 (9.5)	320 (9.8)	31 (23)
2022	24 (13)	322 (9.9)	41 (31)
2023	19 (10)	322 (9.9)	47 (35)
Age groups (years), n (%)			
10	1 (0.5)	11 (0.34)	-
20	3 (1.6)	15 (0.46)	-
30	1 (0.5)	45 (1.4)	-
40	6 (3.2)	149 (4.6)	2 (1.5)
50	20 (11)	296 (9.1)	7 (5.2)
60	17 (8.9)	489 (15)	20 (15)
70	33 (17)	912 (28)	40 (30)
80	60 (32)	942 (29)	35 (26)
90	46 (24)	385 (12)	27 (20)
100	3 (1.6)	16 (0.49)	3 (2.2)
Male, n (%)	115 (61)	1,839 (56)	32 (24)
Settings of death, n (%)			
Out-of-hospital	10 (5.3)	492 (15)	-
In-hospital	49 (26)	2,768 (85)	-
Home or nursing home	55 (29)	-	134 (100)^†^
Missing data	76 (40)	-	-

*Potential corneal donors were defined as individuals who met the criteria of corneal donation but did not donate their corneas for some reason. ^†^Individuals who died in a nursing home were not included in the home cohort of this study.

### OHCA and corneal donation

Table 2 shows the relationship between OHCA and corneal donation. Based on imputed and matched data, the mean number who experienced OHCA cases in the corneal donation group was 26 (13%) out of 190, while the mean number of potential corneal donors who experienced OHCA was 143 (15%) out of 950. OHCA was positively associated with corneal donation after adjustment (odds ratio (OR): 2.2, 95% confidence interval [CI]: 1.3-3.5, p = 0.0019). While the crude OR was 0.89 (95% CI: 0.57-1.4, p = 0.49), this discrepancy likely resulted from the use of multiple imputation and multivariable logistic regression, which accounted for the distribution of matching factors. The robustness of this finding was confirmed in the sensitivity analysis (adjusted OR: 2.2, 95% CI: 1.3-3.8, p = 0.0039).

**Table 2.** Relationship between Out-of-Hospital Cardiac Arrest and Corneal Donation.

	Cases: corneal donation (n = 190)	Matched controls: potential corneal donors (n = 950)	Odds ratio (95% CI)*
Out-of-hospital cardiac arrest, n (%)	26 (13)^†^	143 (15)^†^	2.2 (1.3-3.5)

CI: confidence interval. We matched the case group to the control group at a target ratio of 1:5 using the following matching factors: age group, sex, and period of death. *We performed unconditional logistic regression to estimate the association between out-of-hospital cardiac arrest and corneal donation, adjusting for the matching factors, because matching in case-control studies does not itself eliminate confounding by the matching factors and can potentially introduce confounding even when it did not exist in the source population. ^†^The analysis was performed for each imputed dataset, and the results are presented as pooled estimates of mean values according to Rubin’s rules.

### Potential corneal donors in different settings of death

**Figure 2.**
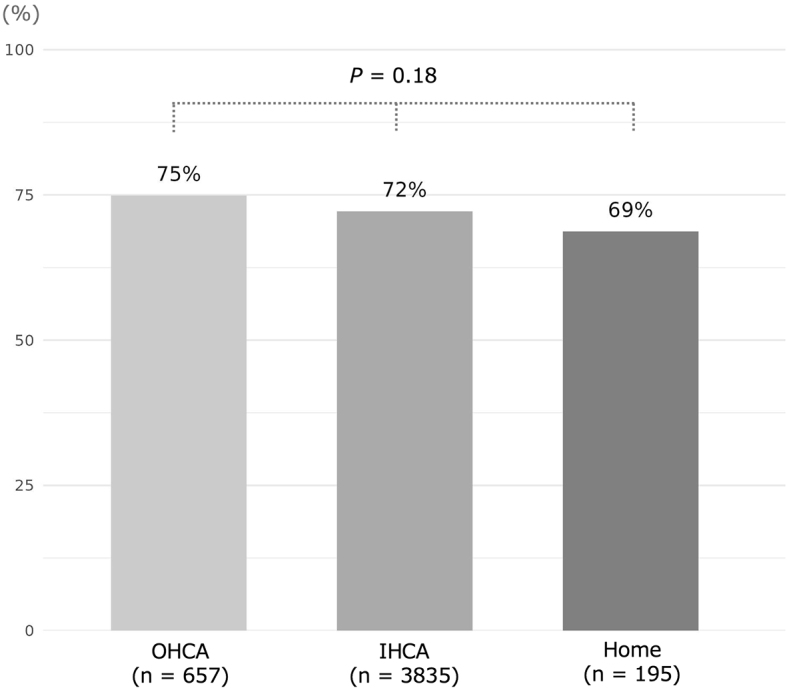
Proportion of potential corneal donors in each setting of death. Individuals who experienced OHCA or IHCA were treated at Hiroshima City Hiroshima Citizens Hospital, and individuals who died at home were treated at Fukui Internal Medicine Clinic. We did not include subjects who donated corneas in each setting of death. These proportions among the three settings of death were compared using the χ^2^-test. IHCA: in-hospital cardiac arrest; OHCA: out-of-hospital cardiac arrest.

Figure 2 shows the proportions of potential corneal donors in different settings of death. Among the combined subjects of the hospital cohort (n = 4,492) and home cohort (n = 195), 72% (3,394/4,687) of subjects were potential corneal donors. The proportion of potential donors who experienced OHCA (75%, 492/657) was the highest among the different settings of death; however, the differences across settings were not observed to be meaningful (p = 0.18). The reasons for ineligibility for corneal donation in subjects who were not potential corneal donors are shown in Supplementary Table S2.

## Discussion

### Summary of findings

Among the 4,492 subjects in the hospital cohort and the 192 subjects in the home cohort, individuals who experienced OHCA, IHCA, or home death were equally likely to be potential corneal donors. Among the 190 individuals who donated corneas in Hiroshima Prefecture, the majority had IHCA or died at home. However, we found that OHCA was positively associated with corneal donation. Individuals with OHCA may be more likely than individuals with other settings of death to donate corneas.

### Possible explanations for the association between OHCA and corneal donation

We hypothesize two primary reasons for the observed positive association between OHCA and corneal donation. First, emergency physicians may be more aware of and proactive about organ and tissue donation issues. In the 2008 Organ Donation Taskforce report, emergency physicians, as well as intensivists, are called upon to play an important role in identifying and referring dying patients who are eligible for organ donation to avoid losing the opportunity for donation after brain death ^(14)^. In other words, emergency physicians require more knowledge and experience in organ and tissue donation than other physicians. Therefore, the results of this study may reflect the attitudes of emergency physicians toward organ and tissue donation. Second, differences in the causes of death in individuals with OHCA and individuals with other settings of death may influence the association with corneal donation. The main causes of death in individuals with OHCA are unexpected deaths, including trauma, sudden cardiac death, acute aortic dissection, and subarachnoid hemorrhage ^(15)^. On the other hand, most of the deaths after IHCA and deaths at home are expected to be due to terminal conditions such as malignancies. Although the feelings of donor families regarding organ donation after brain death are complex, and there is no single reason to accept organ donation, organ donation is more likely to be accepted in cases of brain death due to trauma or stroke than in cases of brain death due to other causes ^(16)^. In cases of corneal donation, the causes of death may also have a greater influence on the acceptability of the option of corneal donation for families of OHCA patients than for families of deceased patients in other settings.

### Clinical implications for emergency physicians

OHCA may be a potential source for corneal donation in Japan. It has been reported that 70% or more of cases of non-traumatic OHCA in Japan result in death in the ED, and these deceased cases are potential corneal donors ^(17)^. In our study, the proportion of potential corneal donors among individuals with OHCA was 75%, and the percentage in a report from an Australian ED was similar ^(18)^. This suggests that approximately 50% of cases of OHCA have the potential for corneal donation. Considering that more than 100,000 cases of OHCA occur annually in Japan ^(5)^, OHCA cases could be an important and adequate source of corneal tissue. The absence of criteria for termination of resuscitation for OHCA in Japan and the distinctive Japanese system of no-charge EMS utilization may contribute to the potential corneal tissue supply. Therefore, targeting individuals who experienced OHCA in the ED to increase corneal donation may help address the persistent issue of a shortage of corneas for transplantation in Japan. This approach would require a routine referral system (RRS) that presents the option of corneal donation to families of patients with OHCA in the ED. A study from the United Kingdom demonstrated that the corneal donation rate increased dramatically from 1% to 36% when such a system was implemented in the ED ^(19)^. Similarly, the introduction of an RRS in various health care settings has reportedly resulted in corneal donation rates of 10%-30% ^(20), (21)^. If we were to implement an RRS for families of patients with OHCA in our ED and conservatively assume a 10% donation rate, this could potentially yield five corneal donors per year (492 potential donors × 10%/9 years). However, this estimated result is insufficient, considering that the number of corneal donors in Hiroshima Prefecture is about 20 per year, and the corneal transplant waiting list is about 60. Therefore, the effectiveness of an RRS for OHCA cases not only in our ED but also in the EDs of Hiroshima Prefecture should be examined.

### Implications for health policy and policymakers

Our findings also have several important implications for policymakers. First, our findings suggest that promoting corneal donation in OHCA patients represents a feasible and meaningful approach. Insurance cards and driver’s licenses in Japan include options for indicating an individual’s willingness to donate organs. National surveys indicate that while 50% of the general public expresses interest in organ transplantation, only 6.7% have formally indicated their willingness to donate ^(22)^. Increasing awareness of the potential for corneal donation, specifically in OHCA patients, could help bridge this gap and enhance the number of corneal donations. Second, incorporating a commitment to corneal donation into the requirements for obtaining a specialty in emergency medicine or for the certification of teaching facilities in emergency medicine would provide substantial benefits. Corneal donation constitutes the final contribution that emergency physicians can make when life-saving interventions for OHCA are unsuccessful. Integrating the option of corneal donation within routine emergency care protocols represents an important step toward increasing corneal donation. Finally, improving the medical system for corneal donation is essential. Currently, eye banks and ophthalmologists serve as the main entities responsible for corneal donation. However, as the practice of corneal donation for patients with OHCA becomes more widespread, emergency physicians should be included in the process. This multidisciplinary collaboration between EDs, ophthalmologists, and eye banks will facilitate the expansion of corneal donation in the future.

### Limitations

Our study has several limitations that should be considered when interpreting the results. First, our case-control study utilized control data from a single tertiary hospital. This may not be fully representative of the broader population in Hiroshima Prefecture as a whole. Our hospital has a larger number of OHCA cases than the number of cases in other hospitals in Hiroshima Prefecture, which may underestimate the results of our study. Second, the hospital cohort and the home cohort used to assess potential donors were also derived from single facilities, potentially limiting the generalizability of our findings on the proportion of potential donors. Third, this study was conducted in Hiroshima Prefecture. Corneal donation is highly dependent on the availability of eye bank services and the cooperation of ophthalmologists, which may vary across prefectures in Japan. For instance, the response to corneal donation in cardiac arrest cases during holidays or nighttime hours may differ by region. Therefore, caution is needed when generalizing these findings to all OHCA cases in Japan. Finally, we were unable to account for all potential confounding factors that might influence the likelihood of corneal donation, such as causes of death, cultural beliefs, or family dynamics. However, it was difficult to obtain this information due to privacy concerns. Further prospective observational studies that can take these confounding factors into account are desirable.

### Conclusions

Our study provides evidence that OHCA was positively associated with corneal donation in Hiroshima Prefecture. The observed association between OHCA and corneal donation suggests that individuals who experience OHCA may be more likely than those in other settings of death to become corneal donors. To confirm our results, a nationwide study using comprehensive data from eye banks in Japan is warranted.

## Article Information

### Conflicts of Interest

None

### Acknowledgement

We thank the Hiroshima Eye Bank for providing data on corneal donations. We also thank the Department of Ophthalmology and Visual Science at Hiroshima University for performing corneal procurement.

### Author Contributions

The conception of the study: Yuji Okazaki, Noritomo Fujisaki, and Yuki Kataoka. The study design: Yuji Okazaki and Yuki Kataoka. Data collection: Yuji Okazaki, Noritomo Fujisaki, Hideto Fukui, and Toshihisa Ichiba. Data analysis: Yuji Okazaki, and Yuki Kataoka, while data interpretation: Yuji Okazaki, Noritomo Fujisaki, Hideto Fukui, Kyungko Huh, Takayuki Otani, Toshihisa Ichiba, Hiroshi Naito, and Yuki Kataoka. The drafting of the manuscript: Yuji Okazaki and Yuki Kataoka. All authors contributed to the revision of the manuscript and approved the final version for submission.

### Approval by Institutional Review Board (IRB)

This study protocol was approved by the Institutional Review Board of Hiroshima City Hiroshima Citizens Hospital (approval number: 2024-35).

## Supplement

Supplemental Tables
