# Supplementary material for: Potential Corneal Donation in Patients with Out-of-hospital Cardiac Arrest: A Case-control Study
Source: JMA J. 2025 Jun 20;8(3):936–43. doi: 10.31662/jmaj.2024-0380 (PMC12328263; doi:10.31662/jmaj.2024-0380)
Supplement: Supplemental Tables [file 2433-3298-8-3-0936-s001.pdf]

Supplemental Table 1. Strengthening the Reporting of Observational Studies in Epidemiology (STROBE) Statement - Checklist of items that should be included in reports of case-control studies

	Item No	Recommendation	Reported page
**Title and abstract**	1	(*a*) Indicate the study's design with a commonly used term in the title or the abstract	1,2
		(*b*) Provide in the abstract an informative and balanced summary of what was done and what was found	1,2
**Introduction**			
Background/rationale	2	Explain the scientific background and rationale for the investigation being reported	3
Objectives	3	State specific objectives, including any prespecified hypotheses	4
**Methods**			
Study design	4	Present key elements of study design early in the paper	4,5
Setting	5	Describe the setting, locations, and relevant dates, including periods of recruitment, exposure, follow-up, and data collection	5,6
Participants	6	(*a*) Give the eligibility criteria, and the sources and methods of case ascertainment and control selection. Give the rationale for the choice of cases and controls	6,7
		(*b*) For matched studies, give matching criteria and the number of controls per case	6,7
Variables	7	Clearly define all outcomes, exposures, predictors, potential confounders, and effect modifiers. Give diagnostic criteria, if applicable	6,7

Data sources/ measurement	8*	For each variable of interest, give sources of data and details of methods of assessment (measurement). Describe comparability of assessment methods if there is more than one group	6,7,8
Bias	9	Describe any efforts to address potential sources of bias	8,9
Study size	10	Explain how the study size was arrived at	8
Quantitative variables	11	Explain how quantitative variables were handled in the analyses. If applicable, describe which groupings were chosen and why	NA
Statistical methods	12	(*a*) Describe all statistical methods, including those used to control for confounding	8,9
		(*b*) Describe any methods used to examine subgroups and interactions	NA
		(*c*) Explain how missing data were addressed	8,9
		(*d*) If applicable, explain how matching of cases and controls was addressed	6,7
		(*e*) Describe any sensitivity analyses	9
**Results**			
Participants	13*	(a) Report numbers of individuals at each stage of study—eg numbers potentially eligible, examined for eligibility, confirmed eligible, included in the study, completing follow-up, and analysed	10
		(b) Give reasons for non-participation at each stage	10
		(c) Consider use of a flow diagram	Figure 1
Descriptive data	14*	(a) Give characteristics of study participants (eg demographic, clinical, social) and information on exposures and potential confounders	10
		(b) Indicate number of participants with missing data for each variable of interest	NA
Outcome data	15*	Report numbers in each exposure category, or summary measures of exposure	10

Main results	16	(*a*) Give unadjusted estimates and, if applicable, confounder-adjusted estimates and their precision (eg, 95% confidence interval). Make clear which confounders were adjusted for and why they were included	11
		(*b*) Report category boundaries when continuous variables were categorized	NA
		(*c*) If relevant, consider translating estimates of relative risk into absolute risk for a meaningful time period	NA
Other analyses	17	Report other analyses done—eg analyses of subgroups and interactions, and sensitivity analyses	11
**Discussion**			
Key results	18	Summarise key results with reference to study objectives	12
Limitations	19	Discuss limitations of the study, taking into account sources of potential bias or imprecision. Discuss both direction and magnitude of any potential bias	16
Interpretation	20	Give a cautious overall interpretation of results considering objectives, limitations, multiplicity of analyses, results from similar studies, and other relevant evidence	12,13
Generalisability	21	Discuss the generalisability (external validity) of the study results	13-16
**Other information**			
Funding	22	Give the source of funding and the role of the funders for the present study and, if applicable, for the original study on which the present article is based	Title page

*Give information separately for cases and controls.

Supplemental Table 2. Criteria for corneal donation in Japan and reasons for ineligibility of corneal donors in each setting of death

	Out-of-hospital cardiac arrest (n = 657)	In-hospital cardiac arrest (n = 3835)	Home death (n = 195)
Ineligibility of corneal donors, n (%)	165 (25)	1067 (28)	61 (31)
Reasons for ineligibility of corneal donors, n (%)			
More than 6 hours having passed since death	101 (61)	1 (0.1)	12 (20)
Unknown cause of death	-	4 (0.4)	-
Active viral encephalitis	-	1 (0.1)	-
Delayed-onset viral encephalitis*	-	-	-
Systemic infections (e.g., bacteria, viruses, or fungi)†	41 (25)	721 (68)	38 (62)
HIV antibody, HTLV-1 antibody, HBs antigen, or HCV antibody-positive	21 (29)	225 (21)	8 (13)
Creutzfeldt-Jakob disease	-	-	-
Leukemia, malignant lymphoma	2 (1.2)	114 (11)	2 (3.3)
Reye's syndrome	-	-	-
Orbital tumor	-	1 (0.1)	1 (1.6)

HIV, human immunodeficiency virus; HTLV-1, human T-lymphotropic virus type 1; HBs, hepatitis B surface; HCV, hepatitis C virus

* Subacute sclerosing panencephalitis and progressive multifocal leukoencephalopathy were included.

† We included coronavirus infection disease that emerged in 2019 (COVID-19).
